# Using a mobile health application to reduce alcohol consumption: a mixed-methods evaluation of the drinkaware track & calculate units application

**DOI:** 10.1186/s12889-017-4358-9

**Published:** 2017-05-17

**Authors:** Sophie Attwood, Hannah Parke, John Larsen, Katie L. Morton

**Affiliations:** 1 Nuffield Health Research Group, Nuffield Health, London, UK; 2 Drinkaware, London, UK; 3 Health and Well-being Services, School of Sport, Health and Applied Science, St. Mary’s University, Twickenham, UK

**Keywords:** Alcohol, Mhealth, Digital health, Mixed-methods

## Abstract

**Background:**

Smartphone applications (“apps”) offer promise as tools to help people monitor and reduce their alcohol consumption. To date, few evaluations of alcohol reduction apps exist, with even fewer considering apps already available to the public. The aim of this study was to evaluate an existing publically available app, designed by Drinkaware, a UK-based alcohol awareness charity.

**Methods:**

We adopted a mixed-methods design, analysing routinely collected app usage data to explore user characteristics and patterns of usage. Following this, in-depth interviews were conducted with a sub-sample of app users to examine perceptions of acceptability, usability and perceived effectiveness, as well as to provide recommendations on how to improve the app.

**Results:**

One hundred nineteen thousand seven hundred thirteen people downloaded and entered data into the app over a 13-month period. High attrition was observed after 1 week. Users who engaged with the app tended to be “high risk” drinkers and to report being motivated “to reduce drinking” at the point of first download. In those who consistently engaged with the app over time, self-reported alcohol consumption levels reduced, with most change occurring in the first week of usage. Our qualitative findings indicate satisfaction with the usability of the app, but mixed feedback was given regarding individual features. Users expressed conflicting views concerning the type of feedback and notifications that the app currently provides. A common preference was expressed for more personalised content.

**Conclusions:**

The Drinkaware app is a useful tool to support behaviour change in individuals who are already motivated and committed to reducing their alcohol consumption. The Drinkaware app would benefit from greater personalisation and tailoring to promote longer term use. This evaluation provides insight into the usability and acceptability of various app features and contains a number of recommendations for improving user satisfaction and the potential effectiveness of apps designed to encourage reductions in alcohol consumption.

**Electronic supplementary material:**

The online version of this article (doi:10.1186/s12889-017-4358-9) contains supplementary material, which is available to authorized users.

## Background

Excessive alcohol consumption is a leading cause of death worldwide [1]. There is now clear evidence that screening and brief intervention to encourage reductions in alcohol consumption prove effective, at least in the short term [2, 3]. Increasingly, digital tools are being used to deliver such interventions, including via the internet or through smart phone applications (“apps”). These newer platforms for intervention delivery offer a number of benefits over and above more traditional approaches, such as face-to-face intervention in clinical settings. For example, not only does near ubiquitous smart phone ownership and internet access mean greater potential for wide intervention reach, but digital tools also allow for discrete delivery of intervention content, high intervention fidelity and can ensure that participant anonymity is retained [4]. Apps in particular also permit recording of alcohol consumption and risk feedback in real time [5]. These are particularly pertinent benefits in the context of alcohol reduction interventions given that accurately determining alcohol consumption levels based on retrospective recall is acknowledged to be challenging, especially given that high levels of intake can interfere with memory formation [6].

Despite widespread availability of alcohol reduction apps on both iTunes and Google Play stores, clear evidence of their effectiveness so far appears lacking [7]. This likely owes to variability in app content and quality [8]. For example, while the behaviour change techniques (BCTs; smallest observable and replicable components of an intervention that have potential to bring about behaviour change) effective in reducing alcohol consumption are known [9], and interventions based on theoretical underpinnings are generally shown to elicit greater effect sizes than those that are not [10], a recent investigation of publically available alcohol reduction apps has demonstrated that very few include all key BCTs [11]. This represents a missed opportunity, not only to optimise the content of these offerings, but also to enhance user engagement given that inclusion of evidence-based content has previously been shown to be an important user preference [11].

The issue of user engagement is particularly relevant to health interventions delivered via app as drop-off in usage is common (e.g. up to 95% of apps are disengaged with after 1 month) [12]. Such low retention rates are problematic from the perspective of intervention delivery as they preclude provision of longer term support, as well as disallowing the follow-up measurement necessary to determine effectiveness. We are, therefore, currently in a position of needing to understand more about exactly what users of alcohol reduction apps are seeking in terms of these offerings, and which features, designs or functionalities appear to promote longer term engagement.

To date, the few available evaluations that explore user preferences in relation to alcohol reduction apps tend to be limited in their generalisability, owing to the recruitment of either small or targeted sample groups (e.g. college age students) [12, 13], or rely on analyses of user ratings of existing publically available apps [11] (which may be biased towards more extreme views) [14]. The findings of these evaluations seem to point towards preferences for personalised content, social networking capability and ease of functionality, especially in terms of entering drinking data [12, 15]. The ability to set own drinking goals is also highly valued [15]. Similarly, in the broader mobile health (mhealth) literature beyond apps alone, comparable conclusions are also drawn; for example, text message based interventions have demonstrated effectiveness in both decreasing intentions to consume alcohol and in actual drinking behaviour [16, 17], with this type of intervention liked by users on the basis of ease and convenience [18]. Other forms of digital intervention, including email, additionally seem to be accepted by recipients, primarily given their scope to tailor and personalise content [16], but may elicit lower levels of actual engagement compared to similar information and BCTs delivered via mobile phone [19].

The present study intends to add to this evidence base by presenting a mixed-methods evaluation of a publically available alcohol reduction app. The app in question was developed by the Drinkaware Trust and is listed on both iTunes and Google Play stores as the “Drinkaware: Track and Calculate Units” app (hereafter, the “the Drinkaware app”). The Drinkaware Trust is an independent UK-based charity which “*aims to reduce alcohol-related harm by helping people make better choices about their drinking*” [20]. While this evaluation is app-specific, lessons learnt in this context may prove valuable to others wishing to develop effective alcohol reduction apps or to improve upon their existing offering.

Through exploratory analyses of routinely captured app usage data, this study aimed to examine typical patterns of app usage over time, and to understand how these patterns differ according to user characteristics, including pre-existing self-reported alcohol consumption levels and user demographics. In addition, we intended to explore app users’ views of the acceptability, usability and potential effectiveness of the Drinkaware app in qualitative interviews with a sub-sample of users. These interviews aimed to explain and augment the findings of the initial quantitative analysis, to further explore how and why users engaged with specific features contained in the app, and to offer recommendations to improve the design of this and similar products.

## Methods

### Study design

Our analysis of the Drinkaware app utilised a mixed-methods approach, specifically, a sequential explanatory design. This involved collection and analysis of quantitative data followed by a collection and analysis of qualitative data. This approach was chosen given that our quantitative data was uncontrolled, meaning that there are a number of limitations in reporting this data in isolation. The findings of the in-depth qualitative data are, therefore, used in the present study to further explain and aid interpretation of the quantitative findings.

### Overview of the Drinkaware app

As outlined by Drinkaware, their app aims to enable users to 1) calculate the units and calories in drinks and to track alcohol consumption over time, 2) gain feedback on how drinking impacts health and to understand trends in drinking patterns, 3) set goals to reduce drinking that are relevant to their lifestyle and to receive supportive notifications when specific achievements are met, and 4) define geographic locations (e.g. a “local bar” or “supermarket”) where users may feel that additional support to regulate alcohol consumption is needed (referred to throughout as drinking “weak spots”). The app then sends users supportive messages when they reach these locations (e.g. “*You are near one of your designated weak spots. Remember, drinking less has many feel-good benefits*”) in an attempt to break alcohol consumption habits.

**Fig. 1:**
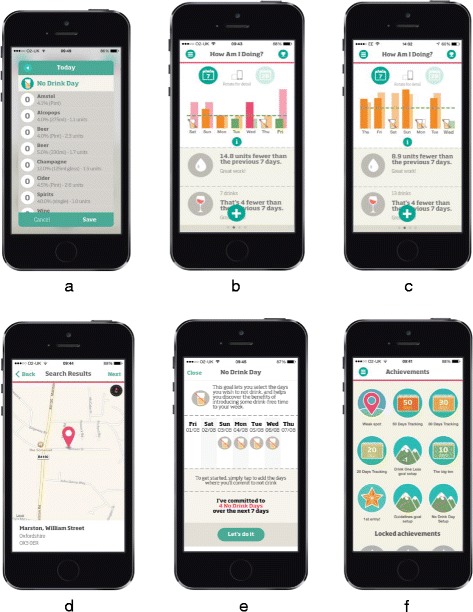
The Drinkaware app **a** Drink entry screen. **b** Drink feedback screen. **c** Dashboard screen. **d** Weak spots screen. **e** Goal setting screen **f** Achievements screen

The Drinkaware app has been publically available since August 2014, and was downloaded by over 170,000 users in the first 12-months following release. Figure 1 provides an overview of the app interface, including the drinks entry screen, risk feedback screen and features comprising the app. Table 1 outlines the specific BCTs incorporated into the app.

**Table 1:** Behaviour Change Techniques included in the Drinkaware app

App Feature	Behaviour Change Technique [21]
Recording the number of alcoholic drinks consumed per day	Self-monitoring of behaviour
Feedback of the number of alcoholic drinks consumed and associated risk level	Feedback on behaviour
	Information about health consequences
Feedback of the cost and calorie equivalent of alcoholic drinks consumed	Feedback on outcomes of behaviour
Setting goals to reduce alcohol consumption (e.g. no drink day, drink within guidelines, drink one less)	Goal setting (behaviour)
	Action Planning
Identification of drinking “weak spots” based on geolocation	Avoidance/ reducing exposure to cues for behaviour
Reductions in alcohol consumption are awarded via notifications	Social reward

### Quantitative phase

#### Data collection

App usage data was collected and analysed over 13-months (between 10th August 2014 and 8th September 2015). Consent to use this data was acquired from all users through a mobile license agreement presented in the app upon first use. Information was collected on user demographic characteristics (age and gender), month of first access, initial motivations for downloading the app (five forced-choice response options including “to reduce drinking”, “to lose weight”, “to be healthier”, “just curious” and an option to provide no information), usage of primary features contained in the app (e.g. goal setting (Fig. 1e), “weak spots” (Fig. 1d) and alcohol consumption self-monitoring and feedback (Fig. 1b)). As Fig. 1a shows, the app interface allowed users to record (for each day of the week) the 1) type (i.e. beer/wine/spirit), 2) measure (e.g. pint, single) and 3) brand of alcohol consumed. Units referred to in the app are based on UK definitions (10 ml or 8 g of pure alcohol). Exploratory analyses of drinking behaviour presented in the results section are based on these self-reported measures only, with no additional alcohol use questionnaires deployed.

#### Data analysis

Data files were first anonymised and imported into excel where they were processed into a format suitable for analysis by statistical package SPSS. Anonymisation involved converting geo-location data from the app, specifying users ‘home’ location, into an Index of Multiple Deprivation (IMD) score (an area-level indicator of socio-economic status) via home postcode. This process was undertaken by the internal research team at Drinkaware who had no involvement in data analysis. No other personal sensitive data were extracted from the app. Only after anonymisation were the data were passed to the external researchers (SA, KM) for analysis purposes.

Given the vast amount of usage data available, and in expectation of a large drop-off in usage within the first month, a decision was made to analyse drinking related outcomes reported at pre-specified time points only. These were during ‘on-boarding’ into the app (i.e. information input into the app on typical weekly alcohol consumption at the point of registration), and then at weeks one, two, three, four and twelve thereafter.

Descriptive statistics were used to summarise user demographic characteristics and to explore usage patterns. From routine data captured, were able to derive the following drinking outcomes: units consumed per week, risk level (e.g. lower, increasing or higher – see Table 2), number of binge drinking session per week (defined as eight or more units per session/day for men, and six or more units per session/day for women) and the number of days in which users indicated that no alcohol was consumed (“no drink days”). We examined differences in these outcomes across user subgroups (i.e. comparing male and female users, younger and older users) using chi-squared and/or one-way ANOVA. Multivariate regression analyses were conducted to explore relationships between user characteristics, usage patterns and drinking behaviour. Paired sample t-tests were used to compare drinking behaviour reported at each time period to the quantity consumed at “baseline” (i.e. at the point of on-boarding).

**Table 2:** Specification of user risk level^a^

**Higher risk:** consumption of more than twice the upper limit of the lower risk daily guidelines (6 units for women, 8 units for men), on four or more occasions within 1 week. Alternatively, consumption of more than 50 units within 1 week for a man, or more than 35 units within 1 week for a woman, regardless of the regularity of drinking
**Increasing risk:** consumption of more than the upper limit of the lower risk daily guidelines (3 units for women, 4 units for men), on four or more occasions during the week. Alternatively, consumption of more than 21 units within 1 week for a man, or more than 14 units within 1 week for a woman, regardless of the regularity of drinking.
**Lower risk:** consumption of more than the upper limit of the lower risk daily guidelines (3 units for women, 4 units for men), on no more than three occasions during the week. Alternatively, consumption of no more than 21 units within 1 week for a man, or no more than 14 within 1 week units for a woman, regardless of the regularity of drinking.

^a^Risk definitions were chosen by the Drinkaware independent Medical Advisory Panel, prior to the new CMO alcohol guidelines published in January 2016 [22] and are based on definitions stated in the Health Survey for England [44]

### Qualitative phase

#### Recruitment

Existing app users who had previously expressed an interest in receiving further information from Drinkaware, and who had provided a contact email address, were invited to complete an anonymous feedback survey. The survey collected information on app usage patterns and user satisfaction (for more details, see Additional file 1). As part of this, users were asked to indicate whether they would be interested to take part in a telephone interview to give more detailed feedback on their experiences using the app.

Maximum variation sampling was used to attempt to interview survey respondents that represented a broad spectrum of app users (i.e. in terms of their initial motivations for downloading the app, demographic characteristics and current app usage patterns). Recruitment criteria included:

- Aged between 18 to 65 years
- Resident in the UK
- Able to consent to and complete a telephone interview in English.

Individuals who agreed to be interviewed were emailed an information sheet and consent form. Following receipt of a signed consent form via post or email, a member of the research team at Drinkaware (JL, HP) who had experience in conducting qualitative research then contacted respondents, by telephone, at a time of their convenience and completed a 30–40 min semi-structured interview. All interviews were audio-recorded, with interviewees verbally consenting to this process prior to the start of the interview. Interviewees were made aware that they were free to withdraw from interviews (and to withdraw disclosed information) at their discretion. Ethics approval for this study was granted by St. Mary’s University, Twickenham ethics committee.

#### Materials

The interview schedule was developed by external researchers KM and SA (see Appendix 1) to probe any questions or issues that arose during the quantitative analysis and to explore users’ experiences of the app, perceptions of usability, acceptability, effectiveness and to obtain suggestions for improvements to this product.

#### Analysis

Framework analysis was used to analyse interview transcripts [21]. This approach was chosen as is permits key themes to be explored across the whole data set, whilst also ensuring that the views of each research participant remain connected to others that they express during interviews, thereby taking into account the wider context surrounding a specific statement.

External researchers KM and SA coded anonymized interview transcripts (50% each) and created themes linking individual codes. To ensure rigour of the coding process, 25% of the interview transcripts were double coded. Decisions on final themes and their constituent codes was an iterative process: using the first five transcripts, an initial analytical framework was co-created by KM and SA, informed in part by the journey that users take through the app (i.e. from download, through the process of “on-boarding” to weekly usage etc.). This ‘analytical framework was refined following discussion with the Drinkaware researchers (HP and JL) and then used by KM and SA to index subsequent transcripts and was expanded as the data demanded. This led to the eventual creation of a detailed analytic framework (developed in excel) summarizing all themes to emerge across the entire data set in a coherent structure. The approach enabled comparison of data across cases as well as within individual cases. Throughout the analysis process, KM and SA kept separate notes detailing their interpretations and other potential themes to emerge.

## Results

### Quantitative findings

#### User demographic characteristics, motivations to download the app and baseline drinking data

**Fig. 2:**
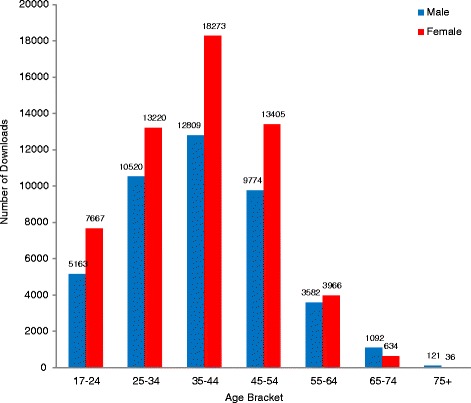
Number of Drinkaware app downloads, by age and gender

In sum, 119,713 individuals downloaded the Drinkaware app. Overall, the app was more frequently downloaded by women than men (*n* = 69,850 users, 59.3%) and by the 35 to 44 age bracket compared to other age brackets (*n* = 31,082 users, 31.0%). This reflects the population targeted by app promotion activity over this period (e.g. women aged 35 years and older). Figure 2 presents details of app downloads, disaggregated by age and gender.

**Fig. 3:**
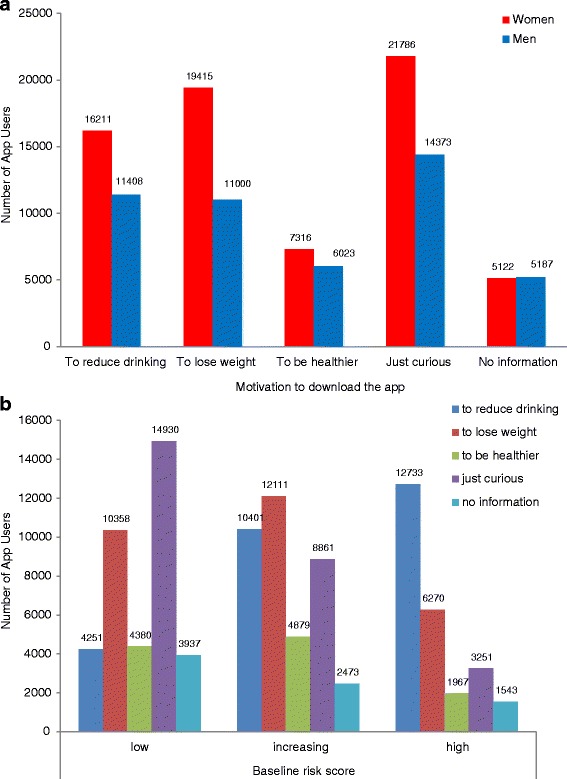
**a** Motivation to download the Drinkaware app, by user gender. **b** Motivation to download the Drinkaware app, by user risk level

During on-boarding, all users were prompted to provide details of their motivations for app use. Of the five forced-choice response options available, “just curious” was most frequently specified by both male (*n* = 14,373 users, 29.9%) and female users (*n* = 21,786, 31.2%), followed by “to lose weight” for female users (*n* = 19,415, 27.8%) and “to reduce drinking” (*n* = 11,408, 23.8%) for male users (Fig. 3a). Gender differences in the proportion of users specifying each motivation were significant (χ2 = 781.07, df = 4, *p* = <0.001).

Considering motivations in relation to alcohol consumption at the point of downloading the app, users classified as “low risk” most frequently stated that they were “just curious” in the app (*n* = 14,930, 39.4%), those at “increasing risk” most commonly wanted “to lose weight” (*n* = 12,111, 31.3%), while “to reduce drinking” was the modal choice for those classified as “high risk” (*n* = 12,733, 49.4%) (Fig. 3b). Differences in motivation preference by baseline risk level were statistically significant (χ^2^ = 13,995.78, df = 8, *p* < 0.001).

Of all users who downloaded the app, 102,367 provided baseline drinking values for a complete week (86.9%). Of these, 22 outliers were excluded from subsequent analyses as these users reported consuming in excess of 300 units per week. In total, therefore, 102,345 users provided valid on-boarding baseline data for further analyses.

On average, male users reported consuming significantly more units of alcohol than female users (34 units per week versus 26 units per week (*t* = 48.34, df = 100,499, *p* < 0.001)). In context, the UK Chief Medical Officer advises consumption of no more than 14 units of alcohol per week to keep associated health risks low [22]. A relatively large proportion of app users (6.8%) also reported “0” unit consumption during a “typical week” at on-boarding. This sample were found to be slightly younger (36.1 compared to 39.1 years) and contained a higher proportion of male users (49.0% compared to 42.8%) than those entering >0 units per week consumed at on-boarding (*p* < 0.001).

Regarding drinking patterns, users self-reported engaging in a drinking “binge”, on average, 1.9 times per week and remained alcohol-free on 3.5 days per week. Approximately one quarter of users were classified as “higher risk” based on self-reported “typical week” consumption at on-boarding (*n* = 10,442 users (24%) of male users, *n* = 14,794 (25.9%) of female users). This compares to only 5% of men and 3% of women in the UK population classified as at “higher risk” based on these definitions [23].

#### Pattern of app use

In those who completed on-boarding, a large drop-off in app usage occurred over the follow-up period. For example, after 1 week the number of app users (i.e. those recording alcohol consumption at least once during the week) dropped to *n* = 51,027 at week one (42.6% of those to download the app). This further reduced to *n* = 17,257 users (14.4%) by week four and *n* = 6025 users (5%) by week twelve.

**Fig. 4:**
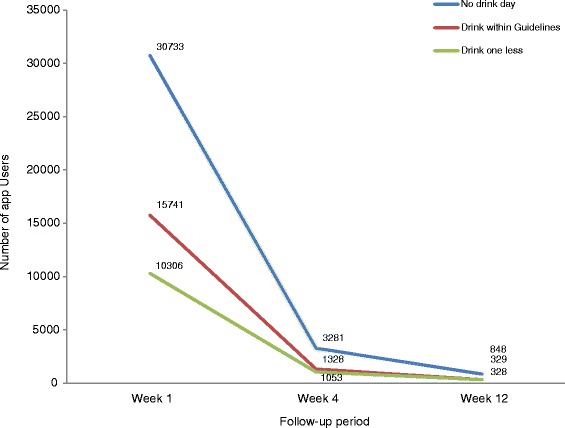
Number of Drinkaware app users setting a new alcohol reduction goal over time

Regarding other main features contained within the Drinkaware app (e.g. goal setting and “weak spots”), approximately 36% of users (*n* = 42,972) set a least one of the three alcohol reduction goals available (“no drink day”, “drink within guidelines” and “drink one less”; see Fig. 1), with goals most frequently set for the first time during the first week of app use. Across the follow-up period, “no drink day” was the most popular goal (see Fig. 4). In general, goals were more frequently set by female than male app users (*n* = 27,682, 39.6%), the 35 to 44 year age bracket compared to other ages brackets (*n* = 14,013, 35.3%) and by those specifically motivated ‘to reduce drinking’ compared to all other motivations (*n* = 17,605, 41.0%). Users who set goals were also more likely to be classified as at “higher risk” at the point of downloading the app than those who did not engage with this feature (e.g. consuming an average of 37 units compared to 25 units in a typical week, *t* = −63.2, df = 87,090.41, *p* < 0.001).

**Fig. 5:**
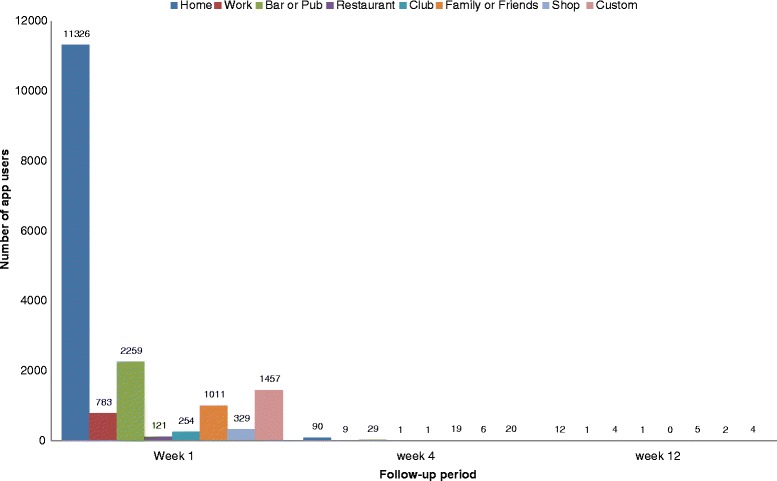
Number of Drinkaware users setting a new “weak spot” over time

A total of *n* = 16,843 (14%) of users chose to define a drinking “weak spot”, with most (*n* = 12,627 users, 75%) specifying just one location (of eight possible categories including “home”, “work”, “bar or pub”, “restaurant”, “club”, “friends’ or family members’ home”, “shop or retailer” or “custom option”). Home was the most commonly chosen “weak spot” at all follow-up time points (see Fig. 5). Once again, “weak spots” were more frequently set by female than male users (*n* = 11,200 users, 66.5%), those aged 35 to 44 years compared to other age brackets (*n* = 5388 users, 34.5%) and those motivated “to reduce drinking” compared to other motivations (*n* = 7754 users 46%). Similarly, users who defined a drinking “weak spot” reported consuming significantly more units of alcohol at on-boarding than those who did not engage with this app feature (e.g. an average of 40 units versus 28 units in a typical week, *t* = −45.18, df = 21,275.08, *p* < 0.001). When examining the number of times a “weak spot” was actually passed and a supportive notification sent to users, zero was most frequent value (e.g. the majority of users appear to have turned off notifications for this app feature).

#### Drinking behaviour over time

Drinking behaviour over time was examined in a subgroup of users who downloaded the app at least 12-weeks before the end of the follow-up period (e.g. those who proved valid drinking data at baseline and who entered the study period in sufficient time to allow for data collection at all follow-up time points; *n* = 73,538). Half of these users entered drinking data during week one (*n* = 37,065 users, 50.4%), with data entry diminishing thereafter. In total, 5045 (6.9%) users entered drinking data at all pre-specified follow-up time points.

We examined drinking patterns of users who consistently engaged with the Drinkaware app over time. These “engaged users” were defined as those entering data on their alcohol consumption, covering at least 6 days per week, for 12-weeks (*n* = 3401 users; 4.6% of the 73,538 users cited above). Compared to the 70,147 users who supplied 12-weeks of data but who did not engage consistently with the app, engaged users were older (42.6 compared to 38.7 years), contained a higher proportion of male to female users (57.4% compared to 43.9%) and reported higher levels of baseline alcohol consumption (31.6 compared to 29.7 units per week; *p* < 0.001 in all cases). Engaged users also most commonly reported “to reduce drinking” as their motivation for using the app (30.6%), compared to non-engaged users who more frequently cited that they were “just curious” (*p* < .001).

**Fig. 6:**
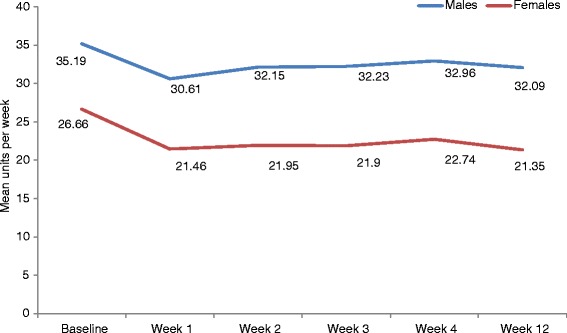
“Engaged” app user average unit consumption over time (*N* = 3401)

A reduction in self-reported unit consumption was shown in engaged users (on-boarding “typical week” = 31.6 units per week; week 1 = 26.7 units; week 4 = 28.6 units; week 12 = 27.8 units; see Fig. 6). A reduction in binge sessions per week was also observed (on-boarding “typical week” = 1.9 binge sessions per week; week 1 = 1.6 per week; week 4 = 1.6 per week; week 12 = 1.6 per week). Finally, the number of “no drink days” per week increased (on-boarding “typical week” = 2.7 days per week; week 1 = 3.3 days per week; week 4 = 3.1 days per week; week 12 = 3.1 days per week). All follow-up values were significantly different to self-reported alcohol consumption at on-boarding when compared using paired samples t-tests (*p* < 0.05). However, no further improvement in drinking behaviour was seen after week 1 (see Fig. 6), with levels appearing to plateau, but remain constant after this point.

#### Exploring determinants of drinking behaviour over 4 weeks in engaged users

In order to explore in greater detail the demographic and motivational characteristics of engaged app users and patterns of usage associated with change in drinking behaviour during the first month of use (i.e. prior to significant drop off in use), multiple linear regression analyses were conducted on the sub-sample of engage users (*n* = 7785). Gender, age, season of download and baseline drinking were entered into the model initially, explaining 28.4% of the variance in unit consumption at 1 month follow-up (adjusted R^2^ = 0.284, *p* = <0.001). Male gender and self-reported alcohol consumption at the point of on-boarding were the only significant predictors in this step. Controlling for the abovementioned variables, user motivation was then entered into the model, explaining an additional 0.004% of the variance in unit consumption at 1 month (adjusted R^2^ = .288, F change = 10.54, *p* < 0.001). Finally, variables that represent usage of different app features were added to the model, explaining an additional 0.002% of the variance in unit consumption at this point (adjusted R^2^ = 0.289, F change = 9.12, *p* < 0.001). Use of goal setting was associated with lower unit consumption at 1 month follow up (1.9 fewer units per week, compared to those who did not use the goal setting feature, *p* < 0.001). Use of the “weak spot” feature did not however predict unit consumption at 1 month (*p* > 0.10).

### Qualitative findings

#### Participant characteristics

A total of *n* = 3491 app users supplied contact email addresses and were sent an anonymised feedback survey. Over the course of a 1 month recruitment period, 189 users completed this survey, 40 of whom agreed to be interviewed. Twenty-one users were recruited to interview. We were unable to recruit more than two interviewees who had downloaded the app, completed on-boarding, but had disengaged directly thereafter (denoted by ID code “A”). We were also unable to recruit more than two interviewees who had used the app to track drinking for a period of time, but had since discontinued usage (denoted by ID code “B”). The majority of interviewees were, therefore, current users of the Drinkaware app (ID code “C”). This indicates that our interview data may not well reflect the views of those who disengaged with this product or who were unavailable or unwilling to consent to participate in research interviews. Interviews were terminated after 19 completed given repetition of themes in this sample. Key participant characteristics are outlined in Table 3 below.

**Table 3:** Characteristics of Drinkaware app users who completed an interview

Interviewee Identifier	Gender	Age bracket (years)	Risk profile	Motivation for downloading the app
A1	Female	25–44	Low risk	To reduce drinking
A2	Female	45–60	High risk	No information
B1	Male	45–60	High risk	To reduce drinking
B2	Female	25–44	Low risk	To be healthier
C1	Male	61–64	Low risk	To reduce drinking
C2	Female	45–60	High risk	To lose weight
C3	Female	25–44	High risk	To be healthier
C4	Female	45–60	Low risk	Just curious
C5	Female	25–44	Low risk	To be healthier
C6	Female	45–60	High risk	To reduce drinking
C7	Female	45–60	High risk	Just curious
C8	Female	25–44	Low risk	To be healthier
C9	Male	25–44	Low risk	To reduce drinking
C10	Female	17–24	High risk	To reduce drinking
C11	Male	25–44	High risk	To reduce drinking
C12	Male	45–60	High risk	Just curious
C13	Male	25–44	High risk	To reduce drinking
C14	Female	45–60	High risk	Just curious
C15	Male	25–44	High risk	No information
C16	Male	25–44	Low risk	Just curious
C17	Male	17–24	High risk	To reduce drinking

### Getting started with the app

#### The on-boarding process

Regarding app usability at the point of on-boarding, the majority of interviewees commented on the “*ease*” of getting started with the app, describing the process as “*intuitive*”. Entering “typical week” alcohol consumption during app registration was designed to be relatively straightforward, yet a number of interviewees indicated uncertainty as to how valid this measure was, mentioning that there was really no true typical week that characterised their drinking:

*“But initially, I thought, this is quite a strange thing to do. And I was thinking back to my week, and thinking, oh God, I can’t remember. I was just throwing some random figures and drinks in there, that I thought were roughly accurate, but I wasn’t completely sure.”*_*(C10)*_

This statement may help to explain the relatively high number of users reporting “0” units consumed at on-boarding. This value either accurately reflects consumption of no alcohol in those who may be seeking the app to assist in maintaining abstinence, or alternatively, represents users who do not wish to, or who are unable to, disclose their typical alcohol consumption levels. This raises questions as to the accuracy of the drinking data obtained during on-boarding as a valid baseline for comparison against further weeks within the quantitative evaluation.

#### Motivations for downloading the app

When questioned about motivations for downloading the app, almost half of interviewees gave multiple motivations. These included motivations not originally covered in the default list incorporated into the app (e.g. to reduce drinking, to lose weight, to be healthier, just curious, no information). For example, interviewees often specified that they wanted “to reduce drinking” in order to achieve a secondary goal (i.e. improve their appearance, feel better, improve their mental health or be healthier in general), or simply that there was no single motivation, but a combination that were relevant to them:

*“It was health reasons, there wasn’t really any one, just one thing, it was a series of things….it’s says it’s no good for your health and then if you don’t have good health then it affects your appearance”*_*(C9)*_

*“I’m not sure if this is sort of a usual thing to download it for, but as well as sort of improving just my physical wellbeing, it was also an experiment for me, to see if it affected my mental wellbeing as well. So the whole reason I actually downloaded it, is because I thought, I wondered if certain difficulties I was experiencing at the time, were linked to excessive drinking. So it was actually more for the mental wellbeing, than the physical wellbeing”*_*(C10)*_

### Patterns of app usage

#### Self-monitoring alcohol consumption

The majority of interviewees reported daily self-monitoring of alcohol consumption, a finding supported by the results of the quantitative analysis (i.e. individuals who did choose to engage with the app after the point of on-boarding tended to then record drinking on most days of the week, rather than just on just one or two sporadic days):

*“I do it daily most of the time. If I haven’t done it for a few days then I will go back and add it in retrospectively. I’m not in a particular routine with it I would say. I don’t know 80 per cent of the time I do it daily but then I might forget for a few days and just go back and do it”.*_*(C8)*_

For most interviewees, daily recording of drinks was done in the evening, with some leaving this until the following morning. A number of interviewees reported a “routine” surrounding app use in general, in which the Drinkaware app had become incorporated:

*“I’d use it in the evening after…like bedtime sort of time”.*_*(B2)*_

*“Because, I’ve got a couple of other little apps that I look at on a daily, not all apps, but a little regime of four or five, you know, I check the weather and I look at my drink app, and various things like that, a little routine, so pretty much daily”*_*(B1)*_

The notion that patterns of recording drinks varied depending on the drinking environment was raised by a couple of interviewees. Specifically, if users reported drinking at home, then they were more inclined to record their consumption drink by drink (in a small number of cases), or to record all drinks later that same evening (in most cases). However, if they were drinking in a public place (i.e. a bar or pub), they were then more likely to record drinks retrospectively:

*“When I say every day, I tend to fill them in the next day. If I’m out having a drink in the pub, I don’t sit there and each time put it into the app. Either when I come home, depending on the time like. If it was after say, midnight, then you’d have to go back a day so I would leave it ‘til the following morning”*_*(C12)*_

Retrospective recording of drinks appeared to create a number of challenges for interviewees to accurately “*fill in the blanks*”, likely affecting the accuracy of information entered into the app, especially on “*heavier nights*”:

*“But often on the heavier nights, I have to guess because I don’t always do it when I’m out. So when I go to the pub or sometimes when I’m not having very many, I can easily remember so I’ll either do it at the time or after. But to be honest, when I’ve had more, I’m guessing roughly at how much I’ve had”.*_*(C17)*_

In terms of potential effectiveness, a number of interviewees commented that the act of concurrent self-monitoring of drinking “*slows it [drinking] down*”. For example, one interviewee commented that the process of recording all drinks made her feel “*guilty*” if she had drunk too much, while others commented that recording drinks provides a sense of “*accountability*”, ultimately facilitating a reduction in the amount consumed. Interestingly, self-monitoring was still considered useful even in cases where drinking had already reduced to zero units:

*“And I think…..even when I stopped all together, there was something still really…something to be said for still logging that, even though I was looking at a whole month and it would just be all green, and it would be like nothing, nothing, nothing, nothing, it was still quite satisfying to log that somewhere, if that makes sense….And then you’ve got somewhere to actually log that and you’ve got something that shows you the progress that you’ve made. And so that’s really, really helpful, and it’s sort of a little motivator for yourself*_*(C10)*_

### App feature usage

#### Goal setting feature experience

As highlighted in the quantitative analysis, the “no drink day” goal was most frequently set by app users. Regarding feature acceptability, interviewees favoured this goal for its simplicity, stating that it fitted easily into their pre-existing pattern of drinking as they were often already trying to incorporate drink-free days into their week. This goal was generally liked for the fact that it was clear to understand and did not involve the effort of estimating exact quantities of alcohol consumed and their associated risk:

*“To an extent I was already doing it, because…I don’t know why, I just was, I thought, right, okay, you can’t drink on a school night, obviously”*_*(C2)*_

In terms of goal setting feature usability, several interviewees who set a “no drink day” goal commented that the process of pre-selecting specific “no drink days” at the start of a week wasn’t necessarily appropriate. Users expressed a wish for greater flexibility, so that goal achievements could be recognised even when an alcohol-free day did not exactly match that pre-specified. Comments also related to the fact that a week, as defined in the app, was not bounded by Monday to Sunday, but started on the day that the app was originally downloaded. This appears to have confused a number of users:

*“… if I happened to have had no drinks on a day that I didn’t set as a no drink day and then it kind of racks up that way, I swear it [the app] gets confused sometimes. My goal is to not drink Monday to Thursday, but say I didn’t drink Sunday to Wednesday I think sometimes it goes, oh yes, congratulations, you’ve met your goal and I’m a bit unclear whether I have or not.”*_*(C8)*_

Fewer interviewees reported setting “drink within guidelines” or “drink on less” goals. In general, decisions regarding consumption appeared to be more clearly categorized into either consuming or not consuming, with degree of consumption not wanting to be considered by users. Interviewees mentioned that once a decision had been made to allow alcohol consumption on a specific day or in a specific setting, they were uncertain as to whether they would be able to recognize if they were drinking one less (as they had no standard benchmark against which to compare this to), or felt that as they had made the initial decision to drink, they wished to do so without having to respect consumption limits.

*“I felt that I probably couldn’t really pick a day when I’d drunk one less or I thought that would be really easy to do because I don’t drink a set amount on a certain day. I don’t like go to the pub on a certain day and think, right, I’ll have three pints today so this time I’ll just have two instead.”*_*(C17)*_

Furthermore, regarding feature usability, interviewees generally preferred to set one goal only. Interviewees suggested that using the app in this way helped them to isolate a single action that could be clearly achieved, and that setting multiple goals was confusing and potentially de-motivating.

*“I only set one goal because I was very keen to kind of remain focused on one thing. I didn’t want to come and get lost in the app using it like a game. You know, I wanted to use it for one very specific thing.....I think I set it to drink probably within guidelines.”*_(C13)_

For interviewees who chose not to set any goals, reasons included wanting to use the app for purposes other than to change their drinking behaviour (i.e. to self-monitor only, or to regulate their current consumption levels), or because they pre-empted their own failure and so did not wish to disappoint themselves in this regard:

*“No, it didn’t appeal - probably because I thought if I put some goals in I’m probably not going to stick to it, which probably makes me sound a bit naughty.”*_*(C7)*_

#### “Weak spot” feature experience

As indicated by our quantitative analysis, far fewer app users engaged with the “weak spot” geo-location feature. In terms of potential effectiveness, this feature appeared to lack relevance to users, with interviewees revealing that they tended not to associate one specific location with drinking and, if they did, this location was often also associated with many other activities (e.g. their home), so lacked identity as a trigger for alcohol consumption:

*“It doesn’t really work for me…There’s not really any one place where I would go to consume alcohol.*_*(C8)*_

Other interviewees more commonly felt that they were triggered to drink at events or social gatherings, by an emotion (e.g. sadness), through feelings of stress, or they felt that their “weak spot” was more likely to be a “weak day”, such as a Friday or during the weekend, when social drinking is a cultural norm in the UK:

*“I think the weak spot is more not a physical place, the weak spot is because you suddenly think, oh god, I’ll go and have a drink.....Yes, your general circumstances are much more accurate, or much more relevant.”*_*(B1)*_

Interestingly, as identified through the quantitative analysis, even if a specific location was set up as a “weak spot”, interviewees did not commonly receive alerts when there, possibly because they had disabled this feature on their phone:

*“Yes. I did. I set up a few. I’m just wondering if it was meant to give me a notification, but I don’t recall ever getting one.”*_*(C10)*_

### Feedback and notifications from the Drinkaware app

Several interviewees commented on the type of notifications and feedback provided by the app. Specifically, a couple of users commented that this feedback had helped them to accurately “*quantify*” their drinking habits. One interviewee commented that the “*stats*” enabled him to compete with himself each week to try and improve his ratings. Indeed, a re-occurring theme across several interviews was the importance of the “*numbers*”:

*“I like the numbers. I like to track stuff and have some figures behind it rather than just like, oh, I’ll go for a run today. I’ll be like, well, I’ll go for a run today but what’s my time from last time and how can I beat it? And I think that’s why this kind of app appeals to me. If I just put the drinks in and it just said you’re drinking too much but didn’t give any numbers behind it, I’d probably delete it within a few days”.*_*(C17)*_

In terms of acceptability of app notifications, one interviewee commented that this feedback made the app less “*passive*” than a previous diary app used for the same purpose, while another commented that it was the combination of self-monitoring and receiving feedback about drinking that was perceived as particularly beneficial:

*“I guess because you were filling out a diary it’s a bit more passive and the fact is that when you enter your figures on the Drinkaware app that first notification and ongoing notifications made you more aware of what you were doing and you are more inclined to do something about it”.*_*(C13)*_

### Type of feedback received

Within the app, users are provided with several forms of feedback on their drinking behaviour, one of which presents the calories consumed that week in alcohol, along with a real food item equivalent (e.g. “burgers”, see Fig. 1). A couple of interviewees commented that this was the most “*surprising*” aspect within the app:

*“The most surprising thing actually was the amount of calories……the calories was something I hadn’t expected, so I think when I’ve looked back on it, that’s probably been quite useful to see.... You know, if you think about having a massive pizza that might be a 1,000 calories, I’d think twice about eating a really big pizza. But having ten pints in any night, I would link it to be unhealthy and costly and feeling a bit bad the next day, but I wouldn’t have thought before that it was also calorific.*_*(C17)*_

Regarding perceived effectiveness of app feedback, interviewees varied greatly in their opinions of how useful this was, with some commenting that calorie feedback is more motivating and informative than general feedback on the amount of units consumed, while others suggested that this information wasn’t specific or personal enough to be relevant:

*“…it just feels a bit too generic. Whereas with the MyFitnessPal it’s very much, you know, one can of Carling you know you’re going to consume, you know, 210 calories and that’s it for Carling. If you drink Carlsberg it could be 220 or, you know”.*_*(C5)*_

In addition, the Drinkaware app also provides users with feedback on the amount of money spent on alcohol in a particular week. Again, some interviewees reported liking this feedback and finding it motivating, while several others commented that this feedback was not relevant for them:

*“I think putting things in terms of money. So when it works out how much you’ve saved. I think that means a lot to people and that could really hit home as well.”*_*(C9)*_

*“The cost for me personally isn’t so much of a driver; it’s much more just about bringing down the level of drinking and about my health as opposed to what I’m spending”.*_*(C11)*_

Almost one third of interviewees commented that notifications from the app had been switched off. Reasons given were that notifications were considered “*annoying*” or generally disliked. In most cases, it was unclear if it was the content of a notification that was annoying or disliked, or just that these users did not wish to receive notifications in general. For example, a couple of interviewees commented that they always turn notifications off for all apps:

*“I probably had it switched off, I tend to switch notifications off.”*_*(B1)*_

A couple of interviewees also stated that they turned off the notifications due to a concern that others would see that these were from Drinkaware, suggesting that privacy is an important user preference:

*“I think because they were just pinging… and I was just thinking, I don’t really want to read this right now. Obviously, and I don’t know whether they do but I guess most people check their phone when something pings in and you can be with your friends and actually maybe you wouldn’t want to be saying to your friends, I’ve just got a notification from Drinkaware”.*_*(C14)*_

### Perceived effectiveness of the Drinkaware app

Although the quantitative findings highlight a general reduction in self-reported alcohol consumption over time in engaged app users only, the uncontrolled nature of this evaluation means that no causality can be attributed (i.e. we are unsure if this reduction is a result of app usage prompting behaviour change, or simply that this change is recorded by the app over the course of the follow-up period). Hence, to know more about the potential impact of the app on drinking behaviour, perceptions of effectiveness were explored in qualitative interviews directly.

Across interviews, a theme was frequently discussed of increased “*awareness*” of how much alcohol is consumed. Several interviewees talked about how the app had helped them to be more conscious of units and/or quantities consumed, even if this awareness did not directly translate into a reduction in drinking. In general, interviewees commented that the app had made them a more “*mindful*” drinker or more “*thoughtful*” about their consumption levels:

*“Because, just like the name of it, Drinkaware, it made me aware of my alcohol intake….rather than sat there with a calculator, which is really boring, nobody wants to do it, all you have to do is write, large glass of wine, boom boom, you’ve got all your statistics there”.*_*(C9)*_

*“Well, I guess like all these things, I was hoping that it would change my behaviour. I think in reality it just made me more thoughtful about how much I’m drinking, which is probably a good thing”.*_*(C17)*_

Several other interviewees referred to an increased awareness of “*binge drinking*” specifically, and discussed how the app had helped them to realize the “*low threshold*” that would place a drinker within this category:

*“So what I’ve learnt about my drinking habits is that probably I can do 5 days a week and not drink but on a Friday or a Saturday or a Saturday and a Sunday then I’ll drink more, so I might have half a bottle of wine on a Saturday and half a bottle of wine on a Sunday, but that almost puts me into, well it puts me into a red; it puts me in towards binge drinking”.*_*(C14)*_

In terms of actual behaviour change, several interviewees stated that the Drinkaware app had proven useful in regulating their alcohol intake through provision of support, enhancing commitment and providing information. This ranged from perceptions that the app had instigated behaviour change, to a view that the app was a useful “*tool*” to enable behaviour change if a user had already decided on this goal. In other words, where interviewees reported that they had already “*made a decision*” or had a “*drive*” to reduce drinking, the app may make this process easier.

*“I attribute a lot of it [reduction in drinking] to the app I guess because it’s a tool isn’t it? I mean obviously I wouldn’t have achieved it without the wish to do something as well personally, the drive to cut down my drinking, but I think that app is a great tool to help you achieve that”.*_*(C11)*_

A couple of other respondents commented that the app provided “*motivation*” and kept them “*on track*”, thus assisting in self-regulation of alcohol consumption over time:

*“It’s very, very easy to just slip back into how you were before or to just, yeah, go on, I’ll just have a another, and things like that. And so it’s nice to have something completely external from that, that sort of just keeps you on track.*_*(C10)*_

Additionally, for a small number of interviewees who were classified as “low risk” at baseline, the app provided “*reassurance*” that their levels of drinking were “*healthy*” or “*fine*”, with one individual commenting that although he had not changed his drinking patterns, this was not his intention:

*“No, no [app has not changed drinking]….having found that I am not drinking to excess or to a bad problematic level, it’s been quite reassuring to know the amount I’m drinking is you might say fine, so I haven’t had to change anything”.*_*(C16)*_

One interviewee explicitly stated that the app had no effect on their alcohol consumption (this individual was “low risk” initially and was not originally motivated “to reduce drinking”), while another stated that they thought the app may have inadvertently prevented engagement with an alternative drink reduction programme that might have proven more effective:

*“To be honest, in a way, I think it’s probably made things worse, because while I might have been interested in pursuing a programme, like, you know, following that and trying to do something about it, it’s just forced me to go off the app, delete it, and forget all about the idea. Do you know what I mean? So, I might even be worse off after the app, rather than better off”.*_*(B1)*_

### Recommendations for improvements to the Drinkaware app

During interviews, factors that may have influenced app usage over time were discussed, and interviewees provided a number of recommendations to encourage longer term use and/or greater acceptability and satisfaction with the Drinkaware app. Indeed, the main criticism that emerged across interviews was the fact that the current iteration does not allow users to easily view their progress in the longer term. Being able to view changes in alcohol consumption over time was considered important, with interviewees recommending that the app include a clear visual summary of longer term trends in drinking:

*“Maybe a graph of the whole 28 days, might be a really good overview.....so you can see if it’s gone down, if it’s gone up across the month, where maybe at what point in the month it goes up and down, and then, yeah, that might be quite helpful”.*_*(C10)*_

Interviewees suggested that mapping longer term consumption patterns to specific days of the week, months or the year or to other calendar events, may also help to facilitate self-monitoring over time:

*“It might give you a real big overview, say if you’ve been doing it for a year, it might give you a whole overview of how you’ve done in that year and how much you’ve saved, and how much…things like that. That might be quite helpful, to really look at it quite broadly.”*_*(C10)*_

Relating to usability, a number of interviewees also appeared dissatisfied with experiences of “losing data”, for example, following an app update or when replacing their device or switching from the Drinkaware website to the app:

*“Because it’s almost like you expect it to be kind of saved on a…because I’ve got the same email address and the same everything…I just literally swapped the device. So I was quite surprised to sort of lose all that. When you’ve done well that was quite gutting.”*_*(C3)*_

Other recommendations included the desire to receive information from the app that is more carefully personalised to current drinking habits and lifestyle. This includes risk feedback tailored to the demographic characteristics of the user:

*“Yeah, not just generically saying you shouldn’t drink because in the long-term it’s bad for you. It’s like, well, tell me something I don’t know. But if it was personalised and said, this is the long-term effect. I mean, I would even go as far as… I know some people wouldn’t like it but I’d even go as far as saying, if you drink this amount, the risk to your health is like minus 3 years off your life.”*_*(C17)*_

Moreover, a number of users mentioned that they wished that the app would provide suggestions for “*replacements*” to drinking, and also include more specific information on techniques that could be employed to help cut down on the amount of alcohol consumed:

*“…but the education side, how do you try and get out of the yellow [indicating ‘increasing risk’]. Like what ways can you actually…how can you change your behaviour rather than just see that you’re in the yellow.”*_*(C5)*_

## Discussion

### Summary of main findings

This mixed-methods evaluation of the Drinkaware app found that the app is disproportionately downloaded by higher risk drinkers (compared to UK averages), with high attrition observed after 1 week. Users who specifically intended to reduce their drinking, or those who self-reported consuming higher levels of alcohol prior to download, were more likely to engage with the app over time (i.e. to record drinks consistently over multiple weeks) and to utilise the main BCTs contained in the app (e.g. setting goals, self-monitoring drinking and setting up “weak spot” alerts).

Based on this uncontrolled quantitative analysis of routinely collected app usage data, we remain unable to conclude with any certainty that the alcohol consumption of engaged users actually reduced as a result of continued interaction with the app. Among users who completed interviews, however, and in line with existing qualitative findings in this area [18], a view was clearly expressed that the Drinkaware app had helped to raise awareness of the amount of alcohol consumed, facilitated self-monitoring of intake and encouraged more mindful drinking. A number of users explicitly stated that the app had facilitated changes in their drinking behaviour. We do, however, acknowledge that these findings are limited in their generalisability and do not necessarily represent the views and usage patterns of non-engaged users or of those not willing to participate in qualitative interviews.

### Main findings in context

We intended to explore how and why users engaged with specific features contained in the Drinkaware app, and to offer recommendations to improve the design of this and similar products. Generally, for those who engaged with the goal setting feature, a preference was voiced for goal options that were simple and that built on existing patterns of behaviour, with the majority of users setting one rather than multiple goals. These findings stand in support of the results of existing evaluations of app based behaviour change interventions [24, 25], including those focussing on alcohol consumption specifically [8]. Additionally, these findings also support text message based interventions in this area; for example, in a recent mobile phone based alcohol reduction intervention, greater decreases in intentions to consume alcohol were found in recipients who clearly set a goal to reduce drinking prior to the intervention [26]. This finding, in combination with the results of our study, imply that any goal setting features incorporated into mhealth offerings like the Drinkaware app may be best viewed as tools to support users to achieve pre-existing health goals, rather than to prompt formation of new goals.

Related to this, our study also indicates that more information on the link between patterns of consumption and potential health harms may need to be included in the Drinkaware app to ensure that “no drink day” goals are not being used to compensate for, or to permit, subsequent binge drinking. Indeed, as a recent randomized controlled trial of a different alcohol reduction app suggests [13], care needs to be taken that content is used as intended, and not as a means to accurately quantify reductions in drinking at one point in time in order to give license to engage in different, yet equally damaging, drinking patterns at a later date [27].

In many cases, users indicated a desire for the app to include options to specify personal goals, while others wanted goal setting to automatically adapt to their progress. This finding fits with a growing body of literature to show that tailoring content to individual needs, and the ability to “learn” from data previously entered, are particularly sought-after features, and may even enhance effectiveness [12, 28]. As is demonstrated in both our study and within the existing literature, alcohol reduction apps, and indeed other forms of digital intervention that include goal setting features, may benefit from functionality that allows users to change their goals over time or in different contexts, so ensuring that the app takes into account deviations from an originally set goal if it ultimately result in the same or similar behavioural outcome [18].

Our analysis of usage of the “weak spot” geolocation feature does, however, emphasize the fact that any such tailoring needs to be thoughtfully designed and implemented; for example, both the quantitative and qualitative findings presented here suggest that highlighting individual physical environment-specific triggers to drinking is not necessarily seen as useful, and was generally poorly understood by users. Interestingly, this finding reflects that of Crane et al. (2015), who also found that alcohol reduction apps that contain features to enable users to identify and restructure environmental triggers to consumption were associated with lower user ratings [11]. Other existing evaluations of apps that contain geolocation features to prompt change in the context of alternative health behaviours (e.g. smoking cessation, reduction in time spent sedentary) do, however, report somewhat more positive responses to geolocation based features, although also document lower than optimal levels of engagement [29, 30].

Taken together, these results imply that conducting a full “behavioural diagnosis” prior to designing an app, whereby a comprehensive understanding of the behaviour in question is arrived at, in addition to an understanding the context in which it is enacted, is necessary to ensure effective tailoring of app content [31]. Indeed, our mixed-methods evaluation reveals that social or emotional triggers may be a better starting point for tailoring app content than identifying geographic “weak spots”, while framing risk-feedback in terms of users’ initial motivations for downloading the app may also prove beneficial (i.e. selectively presenting calorie feedback to those who are motivated “to lose weight”). Tailoring feedback based on individual characteristics in this way, including based on original motivations, is supported in the wider literature [32].

As with many health related apps, drop-off in usage of the Drinkaware app was large during the first month following download [12]. From the routine data analysed, we were unable to follow-up the drinking behaviour of those who downloaded but subsequently disengaged with the app. This represents a missed opportunity given evidence that screening and brief interventions to reduce alcohol related harms (akin to the on-boarding process into the Drinkaware app) prove effective in reducing alcohol consumption in other contexts [33]. Interestingly, interviewees in our study not only discussed the value of longer term self-monitoring to encourage behaviour change, but frequently mentioned that the app had raised awareness, both of the actual amount of alcohol consumed and of the associated health risks. This finding is supported by an existing qualitative study of a similar alcohol reduction app [15], and would suggest that future research may benefit from exploring whether brief, single interventions to reduce alcohol consumption, delivered via app, prompt behaviour change despite subsequent disengagement.

In users who engaged with the app for more than 1 month, the general pattern was to enter drinking data in one session, usually at the start or end of a day. This is a particularly useful finding for those who may be attempting to develop new app features, as it would suggest that these must not rely on real-time data entry (e.g. developing alert systems when a certain consumption limit is reached). Moreover, our qualitative interviews, like others in this area [7], found that discretion in terms of data entry and feedback was particularly important to users. For example, reflecting wider literature in this area [34], interviewees in our study voiced clear concerns about what other people would think if they saw the Drinkaware app icon on their phone or tablet, suggesting that private feedback is desirable.

We note that our quantitative analysis did show a reduction in alcohol consumption between on-boarding and subsequent weeks in users who regularly entered drinking data into the app, although this sample is very small compared to those who originally downloaded the app (i.e. <5% of all users). This finding is also interpreted cautiously given that “typical week” consumption estimates were based on retrospective recall (i.e. at the point of on-boarding), which differs from the diary entry approach adopted thereafter. As existing research shows, individuals’ assessments of the amount that they habitually drink tend to underestimate intake [35]. Hence, our findings may suggest that a maximum change in drinking is observed after one week of app use and maintained thereafter, or that the reduction in consumption seen during week one may be an artefact of the measurement tools used and that the app needs to incorporate more features that are effective in prompting reductions in alcohol intake over time. The next section outlines a number of recommendations to this effect.

### Recommendations

When asked for suggestions for improvements to the app, interviewees commonly mentioned the feedback that it provided. Specifically, comments centred upon a wish for the app to incorporate a visually appealing means to overview trends in consumption over time. For example, frustration was often reported if the app had “lost” data previously input (e.g. as a result of an update), suggesting that more work may need to be conducted to optimise how information is stored, shared and presented between apps and other online dashboards or interfaces [36].

Regarding presenting longer term trends in drinking, many interviewees mentioned that this information would give them a more meaningful way to judge progress, and could be augmented by linking drinking patterns to relevant contextual information to better understand influences on consumption. To achieve this linkage, the Drinkaware app and others like it may benefit from incorporating ecological momentary assessment techniques (EMA) (e.g. ‘real-time’ data capture in natural contexts) [37]. Indeed, a number of studies are already underway to explore the utility of this technique for understanding contextual triggers to alcohol consumption [38, 39]. We do, however, draw attention to the fact that users frequently appeared to switch off notifications from the app, with a number of interviewees suggesting that they found these intrusive - a finding that is also supported within the wider literature on this topic [18]. As such, when developing app features based on EMA, the added benefit of collecting real-time data on determinants of drinking will need to be carefully weighed against data collection burden [40, 41]. In all cases, we recommend that those developing and evaluating apps in this area take into account “APEASE” criteria [31], which highlight the importance of developing digital health interventions that not only contain features that are effective, but also acceptable, practical, affordable, safe and equitable.

An additional recommendation put forward by a number of interviewees was for the app to provide more information on the effect of alcohol on well-being or daily functioning, and to give a broader holistic picture of how drinking can impact individual mood patterns. While favoured by users, it remains to be determined whether provision of this type of information will be effective in prompting actual behaviour change. That this preference was expressed would, however, suggest there are likely to be significant numbers who download alcohol reduction apps not necessarily to reduce drinking directly, but who instead wish to manage a number of non-health related outcomes. Expanding initial “motivation” options present in the Drinkaware app to include improving mental health or well-being may be a good starting point to understand this need more clearly, as would allowing users to specify multiple, possibly interacting motives for downloading this or similar products (e.g., to reduce drinking in order to lose weight and so feel better).

One further, consistent theme to emerge across interviews was that the current version of the Drinkaware app offers little in the way of maintenance support once drinking had successfully reduced. This is a clear limitation given that continued monitoring of consumption (including zero consumption) is documented within the literature as an effective relapse prevention technique [42]. Future alcohol reduction apps should, therefore, ensure that they incorporate BCTS that not only assist current drinkers to reduce consumption levels, but also help abstainers to maintain this over time.

### Strengths and limitations

This analysis of routinely captured app usage data enabled a large sample to be analysed, with our findings complemented by the qualitative study that permitted clarification of data abnormalities and provided further information on app usage patterns. That we studied a sample of existing users, rather than individuals who were recruited into a trial, enhances the ecological validity of our results. This study presents novel insights into how real app users perceived this product, interacted with the features that it contains and explored what is sought by those who actually download alcohol reduction apps.

We do, however, acknowledge a number of limitations to this study: firstly, a reliance on routinely captured app usage data meant that we were unable to draw firm conclusions regarding the effect of app usage on drinking behaviour. This is both due to the observational nature of the quantitative analysis and to the fact that a separate, validated measure of alcohol consumption was not incorporated (i.e. the act of recording drinks served as both a BCT and also an outcome measure in our quantitative evaluation). As such, we recommend that future studies in this area include alternative, validated measures of alcohol consumption, obtained independently of the app itself (e.g., an AUDIT tool could be sent to users at various intervals; see Garnett et al., 2016 for an example of a rigorous evaluation design adopting a separate ‘outcome’ measure) [43]. This may also permit follow-up of drinking behaviour in users who disengage with the app.

Related to this point, we also recognise that conclusions based on analyses of routinely collected data are likely to reflect the structure of the app and app promotion activities run by Drinkaware over the course of the designated follow-up period. For example, we note that the “no drink day” goal was the first option provided to users on a vertical list (see Fig. 1), suggesting that order effects may, to some extent, account for the frequency with which it was chosen. In order to overcome this limitation, it may be beneficial to employ study designs that counterbalance or randomise the order in which app content is presented to users, and to analyse a sample of data collected during a period in which no additional promotional activities are being run.

For the qualitative study, we were unable to recruit more than two interviewees who had since disengaged with the app. Our interviews may therefore underrepresent the views of those who didn’t find the app acceptable or effective. Indeed, this investigation is limited to analysing data provided by those who continued to engage with the app over time, meaning that the results can only be generalised to longer terms users of alcohol reduction apps, and not to those who discontinue usage or who never download these products in the first place.

## Conclusions

The Drinkaware app appears to be a useful tool for raising awareness about drinking and potentially reducing consumption in individuals already committed to making such changes. The evaluation sheds light on issues surrounding usability and acceptability of the various features of the app, and provides recommendations that generally focus on user requirements for greater personalisation and tailoring. Future evaluations should seek to adopt more rigorous designs, including independent outcome measures to draw stronger conclusions about the effectiveness of apps for reducing alcohol consumption.

## Appendix 1

### Interview schedule

**All: Introduction**

- How long ago did you download the app?
- Are you still using the app?
- (If no) How long did you use it for

**Group A**: Those who have completed the on-boarding (‘my typical week’) of the app but no further;

**Group B**: Those who have used the app for 1 week or more but have stopped using the app at the time of the survey.

**Group C**: Those who are current users of the app. The questions asked will be tailored according to the group to which each participant belongs.

**All: Initial motivation to download the app**

- How did you first hear about the Drinkaware app?
- What was your initial motivation for downloading the app?

**All: On-boarding process**

- How did you find the process of getting started with the app (easy/frustrating etc.?)
- How did you find entering your ‘typical week’?
- Do you remember getting feedback on your typical drinking?
- What did you think about the feedback?
- Did it influence your drinking (how, why)?
- Did you initially set any goals for your drinking? What goals did you set? Why? [*Drink one less; Drink within guidelines; Drink free day*]
- Did you set any weak spots? Where? Why?

**B&C: Using the app over time**

- When you first started using the app, how much did you use it (e.g., once per week, every day?)
- What features did you use regularly? Why?
- What did you think about the notifications you received? Did you change the default settings? Why?
- How often did you record your drinks? Where and when? Did you find it helpful? Is there anything that could make this process better for you? Why/why not?

**C: Using the app now**

- How many times per week do you use the app now?
- When do you use the app?
- Which features do you use now? Why these features?
- Do you have any suggestions for improvements to these features?
- Overall, how do you find the app to use?

**All: Impact of using the app**

- Since using the app, have you changed your drinking? How has it changed?
- What do you think about your drinking? Do you want to change your drinking?
- Your motivation to download the app was X. Do you feel the app has helped you to achieve this?
- Do you think you would benefit from using the app longer term? Why/why not?

**C: Willingness to use the app over time**

- Do you plan to continue using the app over the next few months? Why/why not?
- What would make you to use the app more in future?

**All: General Acceptability**

- What do you think about the information provided via the app?
- Had you heard of Drinkaware before using the app?
- Have you ever visited the Drinkaware website
- Since using the app have you accessed any other information or support regarding your drinking? If yes, were you directed through the app? How did you find this?
- Would you recommend the Drinkaware app to a friend – why or why not?

**A & B: Usage cessation**

- Overall, how did you find the app to use?
- Why did you stop using the app?
- Do you still have the app on your phone or have you deleted it?
- Do you think you might ever start using it again in the future?

**All: General phone and app usage**

- Did you use any other way of monitoring your drinking before using the app?
- Are you currently using any other apps to help to improve or monitor your health? What do you think of these/useful features/things you like or don’t like? Is there anything in these apps which you think could improve a drinking app?
- Roughly how many apps do you have on your phone at the moment? How many of those were free? How many paid for?
- Are you currently using any other apps to monitor your drinking?

**All: & finally..**

- Do you have any other comments or suggestions to help us to improve the Drinkaware app?

## Additional file

Additional file 1: Drinkaware App Survey. (DOCX 33 kb)
