# Supplementary material for: Using a mobile health application to reduce alcohol consumption: a mixed-methods evaluation of the drinkaware track & calculate units application
Source: BMC Public Health. 2017 May 17;17:394. doi: 10.1186/s12889-017-4358-9 (PMC5434584; doi:10.1186/s12889-017-4358-9)
Supplement: Additional file 1: — Drinkaware App Survey. (DOCX 33 kb) [file 12889_2017_4358_MOESM1_ESM.docx]

**Drinkaware App Survey**

***Page 1* Your Experience of the Drinkaware App**

Thank you for helping with our survey. It should take around 5minutes, and if you leave your details at the end, you will be entered into a prize draw to win a £50 Amazon voucher. The winner will be selected and notified by email by 23rd October 2015. Please note the prize draw is open to UK residents 18 years or over only.

Route A: Those who completed on-boarding only

Route B: Those who completed 1 week or more but have now stopped

Route C: Current users

1. (A,B,C) When did you first use the Drinkaware app?

In the last week

1 to 2 weeks ago

3 to 4 weeks ago

1 to 2 months ago

3 to 5 months ago

6 or more months ago

1. (A,B,C) How did you find out about the Drinkaware app?

I read about it somewhere (e.g online, in a newspaper or magazine)

Through poster advertising

Through a friend or family member

Through a professional

I found it on an App store

Other (please specify)……………………………………………………… …………………………………………………

1. (A,B,C) What was your main motivation for downloading the app?

To reduce drinking

To lose weight

To be healthier

Just curious

Other (please specify) …………………………………………………………………………………………………………………………

***Page 2* How you use the Drinkaware app**

1. (A,B,C) Have you used the app since first recording your average ‘Typical Week’ drinking?

Yes

No (Identified group A)

***Page 3***

1. (B,C) Have you used the app in the last week?

Yes (Identified group C)

No (Identified group B)

***Page 4***

1. (C) How often do you open up the app?

Every day

Most days

2-3 days per week

Once a week

Less frequently

1. (C) How often do you record your drinking in the app?

*Always/ usually/ sometimes/rarely/ never*

I add each drink individually around the time of drinking

I add my drinks at the end of a drinking occasion/session

I add my drinks at the end of the day

I remember the drinks I’ve had over several days and add them all at once

I add my drinks when the app sends me a reminder to do so

***Page 5***

1. (B) How often did you open up the app?

Every day

Most days

1-3 days per week

A few times per month

Less frequently

1. (B) How often did you record your drinking in the app?

*Please state how often you do each of the following:*

*Always/ usually/ sometimes/rarely/ never*

I added each drink individually around the time of drinking

I added my drinks at the end of a drinking occasion/session

I added my drinks at the end of the day

I remembered the drinks I’ve had over several days and add them all at once

I added my drinks when the app sends me a reminder to do so

***Page 6***

**Onboarding only (A)**

1. (A) Since completing your ‘Typical Week’ on the app, are you drinking more or less?

I’m drinking much more

I’m drinking more

I’m drinking about the same

I’m drinking less

I’m drinking much less

1. (A) Has completing your ‘Typical Week’ on the app made you think about how much you drink?

Yes

No

I don’t know

***Page 7***

**Using the Drinkaware app (A)**

1. (A) Why haven’t you continued using the app?

*Please state your agreement or disagreement with the following statements*

*(Strongly agree, Agree, Neither agree nor disagree, Disagree, Strongly disagree)*

It was too difficult to use

I couldn’t find the time to use it

I kept forgetting to add my drinks

It didn’t make any difference to my drinking

I prefer a different drinking app

It made enough of a difference to my drinking that I no longer needed to use it

Other (write)……………………………………………………………………………………………………………………………………….

1. (A) Do you have any further comments on your reasons for stopping using the Drinkaware app? (free text)

……………………………………………………………………………………………………………………………………….

……………………………………………………………………………………………………………………………………….

……………………………………………………………………………………………………………………………………….

……………………………………………………………………………………………………………………………………….***Page 8***

**Your views on the Drinkaware app (B,C)**

1. (B,C) How easy was it to get started using the app?

*Very easy/ easy/ neither easy nor difficult/ difficult/ very difficult*

1. (B,C) How easy is it to use the app regularly?

*Very easy/ easy/ neither easy nor difficult/ difficult/ very difficult*

1. (B,C) Has using the app helped you to cut back on your drinking?

Yes

No

I don’t know

1. (B,C) Has using the app made you think about how much you drink?

Yes

No

I don’t know

1. (B,C) Which of the following app features have you ***tried***? (Select all that apply)

Goal setting: drink one less

Goal setting: drink within guidelines

Goal setting: no drink day

‘Weak spot’ setting

None of these

1. (B,C) Which of the following app features have you used on a ***regular basis***? (Select all that apply)

Goal setting: drink one less

Goal setting: drink within guidelines

Goal setting: no drink day

‘Weak spot’ setting

None of these

1. (B,C) How helpful did/do you find the reminders sent from the app?

*Extremely helpful*

*Helpful*

*Neither helpful nor unhelpful*

*Unhelpful*

*Extremely unhelpful*

1. (B,C) Would you recommend the Drinkaware app to others?

Yes

No

Don’t know

***Page 9***

1. (B,C) How helpful did you find the app features which you have tried? (only include those tried)

*Extremely helpful/ helpful/ neither helpful nor unhelpful/ unhelpful/ extremely unhelpful*

Goal setting: drink one less

Goal setting: drink within guidelines

Goal setting: no drink day

‘Weak spot’ setting

***Page 10***

**Your views on the Drinkaware app** (B)

1. (B) Why didn’t you continue using the app?

*Please state your agreement or disagreement with the following statements*

*(Strongly agree, Agree, Neither agree nor disagree, Disagree, Strongly disagree)*

It was too difficult to use

I couldn’t find the time to use it

I kept forgetting to add my drinks

It didn’t make any difference to my drinking

It didn’t appeal to me

I prefer a different drinking app

It made enough of a difference to my drinking that I no longer needed to use it

1. (B) Do you have any further comments on your reasons for stopping using the Drinkaware app? (free text)

……………………………………………………………………………………………………………………………………….

……………………………………………………………………………………………………………………………………….

……………………………………………………………………………………………………………………………………….

……………………………………………………………………………………………………………………………………….

***Page 11***

**Further comments (C)**

1. (C) Do you have any further comments about your experience of using the Drinkaware app? (free text)

……………………………………………………………………………………………………………………………………….

……………………………………………………………………………………………………………………………………….

……………………………………………………………………………………………………………………………………….

……………………………………………………………………………………………………………………………………….

***Page 12***

**About you**

*To finish, we just have a few questions, to help give us a better idea of who is using the app. Please note your answers to these questions are just for analysis purposes only. They will remain completely anonymous and confidential.*

*(All questions to all routes A,B,C)*

1. **Are you:** *(note: required, one answer only)*

[Tick boxes]

Male Female

I prefer not to say

1. **What year were you born in? (YYYY)**

*(Note: Answer not required, answer validation between 1900 and 2015)*

[Open text box]

1. **How would you describe your ethnicity?**

*(Note: drop down list, select one only)*

White English/Welsh/Scottish/Northern Irish/British

White Irish

White Gypsy or Irish Traveller

Any other White background

White and Black Caribbean

White and Black African

White and Asian

Any other Mixed/Multiple ethnic background

Indian

Pakistani

Bangladeshi

Chinese

African

Caribbean

Any other Black/African/Caribbean background

Arab

Any other ethnic group

I prefer not to say

***Page 13***

1. **You said your ethnicity was best describes as [piping from above ‘any other…’]**

**please specify:**

*(note: skip logic: if any other, required)*

[open text box]

***Page 14***

1. **How would you describe your employment status?**

*[note: required, Drop down select one only]*

Employed full-time (more than 30 hours)

Employed part-time (less than 30 hours)

Self-employed

Unemployed but looking for a job

Unemployed and not looking for a job / Long-term sick or disabled / Housewife

Retired

Pupil / Student / In full time education

I prefer not to say

1. **What is the highest level of qualification you have?**

*[note: required, Drop down select one only]*

Left school without sitting exams

O Levels/O Grades/GSCE/Standard Grades

A-Levels/Highers

Further Education

Undergraduate degree or higher

I prefer not to say

1. **What is your country of residence?**

*[Drop down select one only, required]*

***Page 15***

1. **[*If UK*] Which region of UK do you live?**

*[Drop down select one only, required]*

North East

North West

Yorkshire and Humberside

West Midlands

East Midlands

East Anglia

South West

South East

Greater London

Wales

Scotland

Northern Ireland

I prefer not to say

***Page 16***

…& finally, we’d like to ask some questions about your drinking habits to help us understand how people with different habits may experience the app

1. How often do you have a drink containing alcohol?

Never

Monthly or less

2-4 times a month

2-3 times a week

4+ times per week

1. How many standard drinks containing alcohol do you have on a typical day when drinking?

1 or 2

3 or 4

5 or 6

7 to 9

10 or more

1. How often do you have six or more drinks on one occasion?

Never

Less than monthly

Monthly

Weekly

Daily or almost daily

1. How often would you say you drink for the following reasons:

|  | Almost never | Some of the time | Half of the time | Most of the time | Almost always |
| --- | --- | --- | --- | --- | --- |
| To forget about your problems |  |  |  |  |  |
| Because it makes social gatherings more fun |  |  |  |  |  |
| To be liked |  |  |  |  |  |

1. Here are some things people have said they do to moderate their drinking. Have you tried any of these?

|  | I have been doing this for a while | I started doing this recently (last two or three months) | I have done this in the past but I no longer do it | I am not doing this but would be willing to do so | I could never see myself doing this |
| --- | --- | --- | --- | --- | --- |
| Stay off alcohol for a fixed period of time |  |  |  |  |  |
| Drink smaller glasses of wine or smaller bottles of beer |  |  |  |  |  |
| Set myself a drinking limit, e.g. just a glass/bottle |  |  |  |  |  |

1. **Are you interested in being contacted for a brief follow-up telephone interview?**

Those selected for an interview will be paid a £20 Amazon voucher for their time. Interviews will be undertaken by November 2015 and will be approximately 30 minutes in duration.

We will contact people based on the survey responses given so that we can learn about different experiences of using the Drinkaware app.

Yes, I am interested in being contacted for a brief follow-up interview

No, please do not contact me for a follow-up interview

1. *(If 39 Yes)* Thanks for your interest in participating in a brief telephone interview. Please provide us with your email address so that, if selected, we can contact you about this.
2. (If 39 *Yes*) Would you also like to be entered into our prize draw for a chance to win a £50 Amazon voucher? The winner will hear from us by 23rd October 2015 and will be notified via email.

Yes, please enter me into the prize draw

No thanks, I don't want to be entered into the prize draw

1. (If 39 *No*) **If you wish to enter our prize draw for a £50 Amazon voucher please enter your email address.**

Note that your contact information will not be linked to the answers you have given today and will only be used for the purpose of the prize draw. The winner will hear from us by 23^rd^ October.

[enter email address] (note validation of email address format)

Many thanks for completing this Drinkaware survey. We will use your feedback to improve the app and ensure we offer the best possible support to people seeking to moderate their drinking.
